# Real-World Efficacy of Biological Therapies in Severe Asthma: A Focus on Small Airways

**DOI:** 10.3390/jcm13195883

**Published:** 2024-10-02

**Authors:** Josuel Ora, Patrizia De Marco, Enrico Motta, Rossella Laitano, Luigino Calzetta, Paola Rogliani

**Affiliations:** 1 Respiratory Medicine, Policlinico Tor Vergata Foundation, 00133 Rome, Italy; josuel.ora@ptvonline.it (J.O.);; 2 Respiratory Medicine, Department of Experimental Medicine, Tor Vergata University, 00133 Rome, Italy; 3 Department of Medicine and Surgery, Respiratory Disease and Lung Function Unit, University of Parma, 43126 Parma, Italy

**Keywords:** severe asthma, small airways, oscillometry, biological therapies

## Abstract

**Background**: Severe asthma is a challenging condition that often resists traditional treatments and requires high-dose inhaled corticosteroids and other controllers to manage uncontrolled symptoms. Recent advances include the use of biologic agents targeting specific inflammation pathways, which have improved symptom control and quality of life, although their effects on small airways remain less understood. **Methods**: This prospective observational study, conducted at Tor Vergata University Hospital in Rome from July 2021 to March 2024, aims to evaluate the efficacy of treatments in patients with uncontrolled severe asthma. It involves baseline assessments and follow-ups at 1 and 3 months post-biological therapy initiation, focusing on both spirometric and non-spirometric (oscillometry) measurements of the small airways to provide a comprehensive evaluation of respiratory function. **Results**: This study, conducted from July 2021 to March 2024, enrolled 40 patients with severe asthma, ultimately analyzing data from 31 participants who underwent biological therapy. The results showed significant improvements in asthma symptoms, the ACT scores increased significantly from visit 1 to visit 2 (*p* = 0.00008) and from visit 1 to visit 3 (*p* = 0.00047), and pulmonary function tests, with notable increases in FEV1 (from visit 1 (74.97 ± 23.43%) to visit 2 (82.96 ± 26.57%, *p* = 0.041) and to visit 3 (88.89 ± 31.41%, *p* = 0.003)) and quality of life scores, and substantial reductions in specific airway resistance and small airway dysfunction markers (the PEF, %pr post-BD showed significant improvement from visit 1 to visit 3 (*p* = 0.012)). However, oscillometric measurements showed no significant changes post-therapy. **Conclusions**: The study concluded that there was an improvement in the small airways measured by non-oscillometric values, without significant improvements in oscillometric parameters. Additionally, a significant improvement in symptoms was observed after the first month of therapy. There was also a significant increase in respiratory function after one to three months of therapy.

## 1. Introduction

Severe asthma is a complex and heterogeneous disease characterized by high morbidity and resistance to conventional treatments. According to the American Thoracic Society (ATS) and the European Respiratory Society (ERS), severe asthma is characterized by the need for high-dose inhaled corticosteroids along with a second controller (and/or systemic corticosteroids) to maintain control, or it remains uncontrolled despite this intensive treatment [1]. Uncontrolled asthma is defined as at least one of the following: poor symptom control, frequent severe exacerbations, serious exacerbations, or airflow limitation [1]. Similarly, the Global Initiative for Asthma (GINA) defines severe asthma as a subset of difficult-to-treat asthma [2]. This refers to asthma that remains uncontrolled despite adherence to maximally optimized high-dose inhaled corticosteroid–long-acting beta-agonist (ICS-LABA) therapy and appropriate management of contributing factors, or asthma that worsens when high-dose treatment is reduced.

The management of severe asthma has significantly advanced with the introduction of biological agents targeting specific inflammatory pathways involved in asthma pathogenesis. Currently, six biologic therapies have been approved for treating patients with severe asthma [3]. These monoclonal antibodies target specific molecules involved in asthma. Immunoglobulin E (anti-IgE, omalizumab), interleukin 5 (anti-IL5, mepolizumab and reslizumab) and its receptor alpha (anti-IL5Rα, benralizumab), interleukin 4 receptor alpha (anti-IL4Rα, dupilumab), and, more recently, thymic stromal lymphopoietin (anti-TSLP, tezepeluma [4], have shown promise in reducing exacerbations and hospitalizations, improving lung function, asthma control and quality of life, and limiting the use of systemic corticosteroids, with a favorable safety profile [3].

However, the impact of these biological agents on the small airways, which are airways with an internal diameter of less than 2 mm, remains inadequately understood. The small airways are critical in the pathophysiology of asthma due to their role in airflow limitation and inflammation. Although asthma affects the entire bronchial tree, the small airways (those with an internal diameter of 2 mm or less) have been identified as the primary site of airflow limitation in both chronic obstructive pulmonary disease (COPD) and asthma [5,6]. Small airway dysfunction (SAD) appears to be linked to an increased number of exacerbations, poorer asthma control, and more severe bronchial hyper-responsiveness [7,8]. Currently, numerous diagnostic techniques are available to evaluate SAD, from non-invasive to minimally invasive or invasive, such as spirometry (forced expiratory flow—FEF25–75%, forced vital capacity—FVC, FVC/slow vital capacity—SVC), impulse or forced oscillometry techniques (IOS or FOT) (resistance at 5–19 hertz (R5–R19/20), reactance (X5), ΔX5in-esp, area of reactance—AX, frequency of resonance—Fres), multiple breath nitrogen washout (MBNW) test or single breath nitrogen washout (SBNW), body plethysmography (residual volume—RV, RV/total lung capacity—TLC), sputum induction, high-resolution computerized tomography (HRCT), 3He-magnetic resonance imaging (MRI), nuclear medicine (scintigraphy, SPECT, PET), and bronchoscopy [9]. Oscillometry is emerging as a valid and sensitive method for diagnosing and monitoring small airway dysfunction [10,11]. This technique measures respiratory impedance, providing detailed information about airway resistance and reactance at different frequencies. Oscillometry is non-invasive, requires minimal patient effort, and can detect changes in the small airways that might be missed by conventional spirometry [12].

Based on studies to date, the prevalence of SAD in asthma appears to be very high, likely 50 percent or higher [13]. In the ATLANTIS study—the largest multinational research demonstrating the impact of SAD on asthma severity—it was found that 91% of asthma patients had SAD, with it being prominently present across all GINA severity steps. [14].

Despite the potential benefits, only a few small studies have specifically investigated the effect of biological agents on small airways in severe asthma [15,16,17,18,19]. These studies have provided preliminary insights but are limited by their small sample sizes and heterogeneous study designs. Therefore, there is a pressing need for more robust and comprehensive research to elucidate the effects of biological agents on small airway function in patients with severe asthma.

The main objective of this study is to evaluate the effect of biological agents on the small airways in patients with severe asthma. By employing oscillometry, we aim to provide a comprehensive assessment of how these therapies impact small airway function, contributing to better understanding and management of severe asthma.

## 2. Materials and Methods

### 2.1. Study Design

This prospective observational study enrolled participants from July 2021 to March 2024, targeting patients with uncontrolled severe asthma managed at the Respiratory Disease Clinic, Tor Vergata University Hospital, Rome. Evaluations were conducted at baseline (prior to initial drug administration) and at subsequent intervals of 1 and 3 months following therapy initiation, covering three assessment points (T0, T1, T3). At T0, consent was obtained and parameters such as comorbidities, exacerbations in the prior year, blood eosinophils, total IgE levels, and a series of pulmonary function tests (including pre- and post-bronchodilation (BD) spirometry, FOT, exhaled nitric oxide fraction (FeNO), and a Six-Minute Walk Test (6MWT)) were assessed. Questionnaires (Modified Medical Research Council Dyspnea Scale—mMRC; Asthma Control Test—ACT; Sino-Nasal Outcome Test-22—SNOT22; 12-Item Short Form Health Survey—SF-12) were also administered. These variables were reviewed at each follow-up visit. The study adhered to the Helsinki Declaration and received ethical approval, on 7 July 2021, by the Independent Ethics Committee, STUDY PROTOCOL Severe Asthma MDB, CLINICAL TRIALS REGISTRY 132.21 P.U.OSS. The complete protocol is described in Supplementary Materials.

### 2.2. Study Population

Inclusion criteria included patients over 18 years old diagnosed with severe asthma as per the 2019 GINA guidelines [2], stable comorbidities not affecting asthma control, eligibility for biological therapy, and signed informed consent. Patients previously treated with biological agents were eligible. Exclusion criteria were under 18 years of age, active oncological diseases, psychiatric comorbidities, pregnancy, and inadequate availability for follow-up visits.

Normal lung function was defined as FEV1 or FVC values greater than 80% of the predicted value, with an FEV1/FVC ratio above the lower limit of normal. Additionally, FeNO was considered indicative of bronchial inflammation when levels were ≥25 ppb.

### 2.3. Methods

Functional assessments included spirometry (specifically after the administration of 400 mcg of Salbutamol, conducted only during the first (V1) and third (V3) visits), FOT, diffusing capacity for carbon monoxide (DL’co), and the 6MWT. We employed various devices for these tests: the Carefusion MasterScreen PFT (pulmonary function testing) System or the Cosmed Q-Box Body Plethysmography for complete lung function assessment. Respiratory resistance was measured using the ResTech Resmon Pro Full (Version 3). Exhaled nitric oxide levels were assessed using the Bosch Healthcare Vivatmo Pro device (Bosch Healthcare Solutions GmbH, Waiblingen, Germany). Quality of life and symptom evaluations were conducted using several questionnaires: the ACT, SNOT22, mMRC, AQLQ, and SF-12. Measurements were performed in accordance with international guidelines, with further details provided in the Supplementary Materials. The questionnaires used are also described in the Supplementary Materials.

### 2.4. Statistical Analysis

Data were collected in a de-identified database. Continuous variables were checked for normality using the Shapiro–Wilk test, and missing data were managed by excluding incomplete observations from the regression analysis. Descriptive statistics (mean, median, standard deviation) were calculated, and Wilcoxon signed-rank tests were used for paired comparisons. Multiple regression analyses were performed to identify predictors of treatment response, and repeated measures ANOVA was used to assess group differences over time. A formal sample size calculation was not performed due to the limited availability of prior studies and the exploratory nature of the analysis. All statistical analyses were conducted using Python 3 (Pandas: 1.3.3 SciPy: 1.7.1) (Pandas, SciPy) and GraphPad Prism 9, with a *p*-value < 0.05 considered significant (further details are provided in Supplementary Materials).

## 3. Results

### 3.1. Subjects

**Figure 1:**
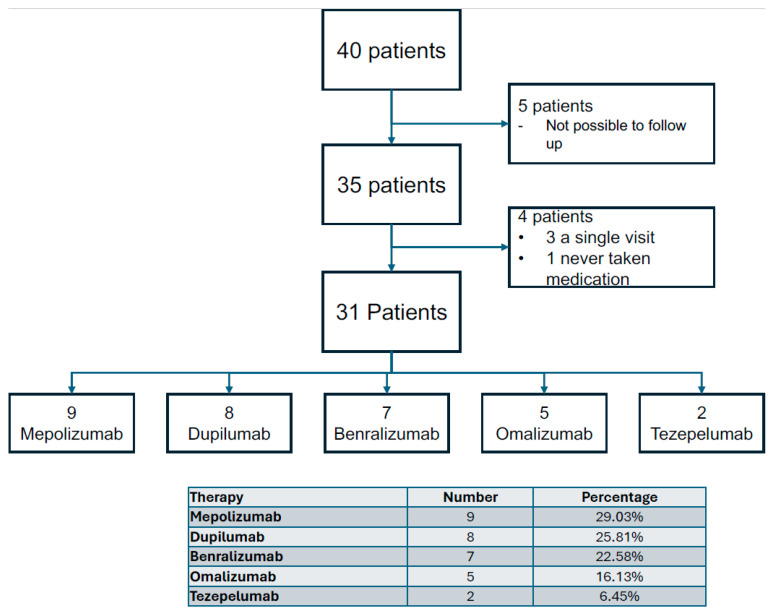
Patient distribution and treatment allocation: clinical flowchart detailing the enrollment and retention of patients in a study evaluating the efficacy of biological therapies for severe asthma.

From July 2021 to March 2024, 40 new patients were enrolled for biological therapy for severe asthma. Of these, 5 were excluded due to irregular follow-up availability. Among the remaining 35, 3 patients initiated biological therapy but completed only the first visit and were thus not included in the final analysis; 1 patient attended the initial visit but did not commence the prescribed therapy. The final analysis included 31 patients, distributed as follows: 9 patients received mepolizumab, 8 received dupilumab, 7 received benralizumab, 5 received omalizumab, and 2 received Tezepelumab (Figure 1). Notably, 3 patients had previously used a different biological drug and switched after an adequate wash-out period, specifically from omalizumab to benralizumab, mepolizumab to dupilumab, and dupilumab to benralizumab. The study population was mostly female (68%), with a mean age of 61.19 years (±13.81). The average weight was 71.06 kg (±16.56), height was 1.63 m (SD = 0.09), and BMI was 26.73 kg/m^2^ (±6.12). The average number of exacerbations per year was 3.35 (±3.32) without hospitalizations (Table 1). The basal characteristics and comorbidities are described in Table 1 and Supplementary Materials.

### 3.2. Questionnaire

The administration of a biological agent improved the symptoms of the patients. The ACT scores increased significantly from visit 1 to visit 2 (*p* = 0.00008) and from visit 1 to visit 3 (*p* = 0.00047), with no significant change between visit 2 and visit 3 (*p* = 0.916). The AQLQ scores improved significantly from visit 1 to visit 2 (*p* = 0.026), but the changes from visit 1 to visit 3 (*p* = 0.108) and from visit 2 to visit 3 (*p* = 0.832) were not significant. The mMRC Dyspnea Scale did not show significant changes across the visits. The SNOT22 scores decreased significantly from visit 1 to visit 2 (*p* = 0.007), with a marginal change from visit 1 to visit 3 (*p* = 0.054), and no significant change from visit 2 to visit 3 (*p* = 1.000). The SF-12 Health Survey scores showed significant improvement from visit 1 to visit 2 (*p* = 0.001) and from visit 1 to visit 3 (*p* = 0.009), with no significant change between visit 2 and visit 3 (*p* = 0.581).

### 3.3. Pulmonary Function Test

The PFTs demonstrated significant improvements following the administration of a biological agent. There were notable enhancements in the percentage predicted values of SVC and FVC pre- and post-BD that were significant just after 3 months, the forced expiratory volume in 1 s (FEV1preBD) improved significantly from visit 1 (74.97 ± 23.43%) to visit 2 (82.96 ± 26.57%, *p* = 0.041) and to visit 3 (88.89 ± 31.41%, *p* = 0.003), with a significant difference between visit 2 and visit 3 (*p* = 0.030). The FEV1/FVC ratio showed significant improvement after three months (see Table 2 for detailed results).

The static lung volumes—total lung volume (TLC) and residual volume (RV)—did not show significant changes across the visits (see Table 3). However, there were notable improvements in peak expiratory flow (PEF) and FEF25–75 (see Table 3).

The PEF %pr pre-BD significantly increased from visit 1 to visit 2 (*p* = 0.017) and from visit 1 to visit 3 (*p* = 0.039). Similarly, the PEF, %pr post-BD showed significant improvement from visit 1 to visit 3 (*p* = 0.012). FEF25–75 showed a significant increase in both absolute values and percentage predicted from visit 1 to visit 3 (*p* = 0.001 and *p* = 0.004, respectively). Post-BD FEF25–75 also increased significantly from visit 1 to visit 3 (*p* = 0.015 for absolute values and *p* = 0.035 for percentage predicted). Specific airway resistance (sRAW) demonstrated significant improvement from visit 1 to visit 3 (*p* = 0.013 for absolute values and *p* = 0.040 for percentage predicted).

### 3.4. Forced Oscillometry Technique Variables

There were no significant differences in the oscillometric variables after therapy (see Table 4). Respiratory resistance at 5 Hz (Rrs5) total and the corresponding percentage predicted (%pr) did not show significant changes across visits. The resistance difference between 5 Hz and 19 Hz (R5–19) increased significantly from visit 1 to visit 2 (*p* = 0.009), but not between visit 2 and 3 or visit 1 and 3. Total reactance at 5 Hz (Xrs5) and its percentage predicted (%pr) did not show significant changes. The area under the reactance curve (AX) and its percentage predicted (%pr) also did not demonstrate significant changes across visits. Resonant frequency (Fres) and its percentage predicted (%pr) showed no significant differences across the visits. The external force length (EFL Xrs) remained relatively stable without significant changes.

### 3.5. Prevalence of Small Airways Disease and Response to Therapy

The analysis of the prevalence of small airways disease in the studied group showed significant variability depending on the measurement method used (Table 5). The prevalence was higher when measured with the FEF25–75 and lower with some oscillometric variables. Moreover, significant changes were observed in several parameters: the proportion of subjects with FVC < 80% showed a highly significant decrease (*p* = 0.0017). There was also a statistically significant reduction in the proportion of subjects with SVC < 80% (*p* = 0.0274). A significant decrease was noted in the proportion of subjects with SVC–FVC < 0.100 (*p* = 0.0071). Additionally, the proportion of subjects with FEF25–75 < 80% demonstrated a very significant reduction after biological therapy (*p* < 0.0001).

In contrast, the following parameters did not show statistically significant changes: the proportion of subjects with RV%pr > 120%pr (*p* = 0.0873), RV/TLC > 0.40 (*p* = 0.5094), sRAW%pr > 120%pr (*p* = 0.2475), Rrs5 tot pre Z score > 1.645 (*p* = 0.6142), Xrs5 tot pre Z score < −1.645 (*p* = 0.6142), AX Z score < −1.645 (*p* = 1.0000), and Fres Z score < −1.645 (*p* = 0.7706).

## 4. Discussion

This prospective, non-interventional study, conducted in a real-life clinical setting, analyzed a cohort of 31 patients with bronchial asthma treated with biological therapies from July 2021 to March 2024. The key findings indicate that biological therapies significantly improved patient symptoms within one month, as assessed by standardized questionnaires (mMRC, ACT, AQLQ, and SNOT22). Respiratory function showed progressive improvement across visits, both pre- and post-BD. However, static lung volumes did not show significant improvements. Small airways involvement varied depending on the indices considered. In our study, oscillometric values did not improve, with the R5-R19 value worsening from visit 1 to visit 2 (R5–19 pre: from 0.48 ± 0.71 cmH_2_O (l/s) at V1 to 0.77 ± 0.73 at V2). The most altered values in the subjects analyzed were FEF25/75% < 80 with a prevalence of 93.55% and RV/TLC > 0.40 with a prevalence of 70.97%, indicating widespread damage to the small airways among patients. Oscillometric alterations, such as Rrs5 tot pre Z score > 1.645 and Xrs5 tot pre Z score < −1.645, were observed in 22.58% of patients for both parameters, showing that only a small percentage of patients exhibit alterations measured by this methodology.

After biological treatment, there was a significant improvement in quality of life (SF-12) and symptoms (ACT, SNOT22, and AQLQ) within the first month. This improvement was both statistically and clinically significant, consistent with findings from previous randomized controlled trials (RCTs) [3].

Our study confirms that the use of biological therapies improves various spirometric parameters. One month after the introduction of the treatment (V2), significant increases were recorded in FVC, with a difference of 0.22 L, and a further increase at three months of 0.47 L. FEV1 followed a similar trend, with an increase of 0.20 L after one month, and an additional increase of 0.33 L at three months. These results suggest a positive pulmonary response to therapy, exceeding what would be expected based on literature data [3,20]. For example, a meta-analysis of 31 studies, including 10,323 participants (5551 on biologics), found that biologics improved FEV1 by 0.11 L [3]. Patients with eosinophil counts ≥300 cells/μL had a greater improvement (0.18 L vs. 0.07 L for counts <300) [3]. In two studies on dupilumab, patients with higher FeNO levels (≥50 ppb) also showed greater FEV1 improvements (0.37 L vs. 0.13 L vs. 0.11 L for lower FeNO levels) [21,22]. Another meta-analysis of 42 trials, including 17,965 patients, showed similar results, where biological agents improved lung function in T2 patients by an average of 0.23 L, with varying degrees of improvement among biologics, but no significant improvement in patients without T2 inflammation [20].

Conversely, static lung volumes did not show significant changes, indicating that treatments did not significantly impact hyperinflation and air trapping. The subjects studied did not show any significant reduction in TLC, RV, and RV/TLC. Compared to the literature, these data are controversial because some studies have shown an improvement in both measurements [23], others only in RV [24], and some have shown no improvements [25].

Similarly, the evaluation of small airways through forced oscillometry did not yield significant results. Key oscillometric parameters, including Rrs at 5 Hz, Xrs at 5 Hz, AX, Fres, and EFL, did not show significant changes between the initial visit and subsequent visits. The only significant finding was the R5-19 pre value between V1 and V2 (*p* = 0.009), indicating a potential treatment effect on the small airways. However, this effect was not sustained at three months. In fact, the increase in this value indicated a worsening rather than an improvement, contrary to expectations.

On the other hand, other indices of small airways disease [9] such as FEF25-75, the difference between SVC and FVC, FVC, and SVC improved after therapy. The prevalence of small airway damage in the studied population was studied, and it was observed that the presence of oscillometric alterations in our population varied between 6.45% and 22.58%, showing a low prevalence. Other indices, such as FEF 25–75% of the pulmonary volume < 80% predicted or the ratio of residual volume to total lung capacity (RV/TLC) > 0.40, showed a prevalence of 71–94%.

Despite affecting the entire bronchial tree, asthma has been recognized to cause airflow limitation primarily in the small airways—those less than or equal to 2 mm in diameter—in both COPD and asthma [26,27]. Overall, the prevalence of SAD in patients with asthma is approximately 50–60%, but it appears to vary depending on the physiological measures used to evaluate it [14]. In the large multinational ATLANTIS study—the most comprehensive investigation to date on the role of SAD in asthma severity—SAD was found to be strongly present across all levels of GINA severity [14,28]. Despite considerable variation in the prevalence of SAD depending on the physiological variables used for its assessment, it consistently remains higher among patients with more severe asthma (GINA step 5) [14].

Investigating small airways is challenging due to the absence of standardized and universally accepted measurement methods, often confining their evaluation to experimental and investigative stages [9,29,30]. Conventional spirometry mainly reflects the variability and reversibility of airway obstruction, making it an imperfect tool for sensitively assessing small airways since abnormalities become evident only when approximately 75% of these airways are obstructed [31]. In daily clinical practice, forced expiratory flow at 25–75% of the pulmonary volume (FEF25–75) is the traditional spirometric index used to assess peripheral airway obstruction. Some studies indicate that FEF25–75 is associated with poorer asthma control and negative asthma outcomes [28,32,33]. Siroux et al. have shown that obstruction of small airways, assessed based on FEF25–75, may contribute to the long-term persistence of asthma and subsequent risk of negative asthma outcomes, regardless of the effects of large airways [33]. However, the utility of FEF25–75% predicted as a marker for peripheral airway obstruction has been challenged by several studies, thus reducing its reliability for assessing SAD [9].

On the other hand, the use of oscillometry for assessing small airways is becoming increasingly widespread [7,10,11,12]. Clinical interpretation of measurements typically relies on the two components of respiratory impedance Zrs, respiratory reactance (Xrs) and respiratory resistance (Rrs) [10,11]. Both Xrs and Rrs, which reflect overall pulmonary impedance, are measured in real time as functions of airflow, volume, and pressure [10,11]. Parameters such as R5 and R20 (or R19) are employed to assess total and proximal airway resistance, respectively [10,11]. Consequently, the contribution of distal airways is determined by the difference between R5 and R20 or R5 and 19, serving as an index of peripheral airway resistance. This method has been applied to asthmatic patients in clinical studies and hospital settings, with a conventional cutoff of R5–R20 > 0.07 kPa·s·L^−1^ (a conservative upper limit for R5–R20) used to define the presence of small airway dysfunction (SAD) [14]. The ATLANTIS study categorized SAD into two clinically significant groups using impulse oscillometry (IOS) and spirometry [14]. Interestingly, this study identified R5–R20 as the IOS-measured marker that most strongly correlated with SAD among various physiological markers of small airways.

Our study data partially diverge from the literature in that although damage to small airways is present in over 90% of patients, FEF25/75 and RV/TLC appear to be the best indicators. In the ATLANTIS study, the prevalence of small airway disease varied depending on the physiological measure used. It was lower with Sacin (19%) and residual volume (RV)/TLC (22%), higher with R5–R20 (42%), and with FEF25–75 [14]. It was hypothesized that this was due to different subtypes of small airway disease, depending on their location in the bronchial tree, with Sacin and RV/TLC reflecting the more peripheral airways and R5–R20, FEF25–75, and the decrease in FVC during PC20 with methacholine reflecting the small and medium-sized airways. However, the main drawback is that it has never been directly investigated which compartment of the bronchial tree each of the aforementioned tests reflects. Our data confirm that the assessment of small airway damage depends on the measurement used; furthermore, the improvement in FEF25-75 and the resistances, like the difference between SVC and FVC during the visits, demonstrate that biological drugs are also effective in treating small airways. What is not confirmed is the presence of an improvement in the indices of FOT. On the contrary, the transient worsening of the R5–R19 index seems to almost testify to a worsening in some patients of the damage to the small airways that most likely reflects rather a delatentization of damage after therapy. It has been hypothesized, though without direct evidence, that reducing inflammation in the large and medium airways may have inadvertently exaggerated the difference between R19 and R5, potentially creating the appearance of worsening airway damage. Further studies will be needed to confirm this hypothesis or to assess whether it is just a data point without any clinical importance. It should be noted that in our study FOT and not IOS, which was used in other studies, was used, and although theoretically these two methods should be equivalent, the relationship between IOS/FOT is not clear, thus making it difficult to compare measurements taken from different devices. In the literature, there are few real-life retrospective or prospective studies that have investigated the effect of biological drugs on small airways [8]. A retrospective study investigating patients with mild-to-moderate asthma and concomitant chronic rhinosinusitis with nasal polyps (CRSwNP) did not observe significant improvements in X5, R5, and AX after 12 months of therapy with dupilumab [34]. There are no data at one month. However, it is important to note that these patients did not present SAD at baseline, and therefore, presumably there would be no room for improvement per se. In a prospective study (n = 18), it was shown that low-frequency oscillometry reactance like X5, a measure of peripheral pulmonary compliance, significantly improved by 74% one month after therapy with mepolizumab in severe eosinophilic asthma [19]. However, another retrospective study on severe asthmatics (n = 30) showed no improvements in R5–R20 or AX after 10 months of mepolizumab [35].

These findings, along with the results of our study, highlight a significant dichotomy: on one hand, FEF25–75 proves to be an extremely sensitive indicator to biological therapeutic interventions; on the other hand, the role of oscillometry and its parameters appears significantly more uncertain, with the scientific literature presenting a series of contradictory results [15,16,18,35]. Our study, while documenting a statistically significant increase in values in some parameters associated with small airways—an increase that, along with the improvement recorded in spirometry and clinical symptoms, undoubtedly attests to the efficacy of the pharmacological treatments under examination on such a population—did not detect any significant effect on oscillometric values. This absence of effect is due primarily to the low prevalence of small airway damage measured with oscillometry, different from other parameters like FEF 25–75, which have proven more effective in measuring it. This discrepancy when compared to other spirometric indicators raises relevant questions, such as the real diagnostic meaning of FOT compared to other spirometric indices, and especially if measurements of different small airway damage may have different clinical meanings. It is interesting to note that oscillometry only refers to current volume in a speculative manner and highlights damage present during the current volume differently, for example, from other indices like FEF25–75 or the difference between SVC and FVC, which instead involve all dynamic pulmonary volumes. One last observation concerns the difference between FOT and IOS, which are two methodologies that are based on comparable physiological principles and aim to analyze the same functional parameters, albeit with two different techniques. Most studies in the literature have used IOS as it historically precedes FOT, while this study uses FOT, and this raises the need for comparative studies on asthmatic patients to assess the interchangeability and consistency of the data collected through these two diagnostic techniques in the context of the evaluation of small airway diseases.

Limitations: The primary limitations of this study include a restricted sample size, which may affect the representativeness of the results and the generalizability of the conclusions. Additionally, data loss due to some patients not completing the study poses a significant challenge, as it could impact the analysis and validity of the findings. The monocentric nature of the study, although ensuring greater data uniformity, might limit the variety of observed conditions and treatments.

Furthermore, the variation in biological treatments received by patients adds another layer of complexity. Since patients were subjected to different biological drugs, it is difficult to attribute observed effects to a specific treatment or a combination thereof. This variation could confound the interpretation of results and may require additional methodological approaches to adequately analyze the impact of each treatment.

Moreover, the limited sample size does not allow for specific analyses on each biological agent used. This limitation could affect the study’s ability to provide detailed insights into the efficacy and safety of each biological treatment in the context of severe asthma. These considerations must be kept in mind when interpreting the study results and might necessitate further research or methodological approaches to mitigate or overcome these limitations.

## 5. Conclusions

In conclusion, this prospective study has demonstrated the effectiveness of biological therapies in significantly improving both symptoms and respiratory function in patients with severe bronchial asthma. Following the initiation of treatment, a rapid and substantial improvement in quality of life, symptoms, and spirometric parameters was observed. Although no significant changes were noted in static lung volumes, the results indicate a general improvement in quality of life and asthma control. However, oscillometric values and measurements related to small airway damage show that not all patients respond uniformly, suggesting a need for further personalization of treatment. These findings underscore the importance of continuing research and optimizing therapeutic strategies to effectively treat bronchial asthma in all its manifestations and complications. Further studies are necessary to confirm these findings and explore the potential individual variability in response to biological therapy, highlighting the need for tailored therapeutic approaches based on detailed phenotypic and possibly genotypic patient characteristics.

## Figures and Tables

**Table 1:** Demographic and clinical characteristics of the study population.

Variable	Mean ± SD (95% Lower CI–95% Upper CI)
Age, years	61.19 ± 13.81 (56.33–66.06)
Weight, Kg	71.06 ± 16.56 (65.23–76.89)
Height, m	1.63 ± 0.09 (1.60–1.67)
BMI, kg/m^2^	26.73 ± 6.12 (24.58–28.89)
Hospitalizations (12 months), n	0 ± 0
Exacerbations (12 months), n	3.35 ± 3.32 (2.19–4.52)
Eos, %	9.16 ± 9.82 (5.45–12.86)
Eos, cells/µL	907.50 ± 1901.06 (203.35–1611.65)
IgE, IU/mL	411.87 ± 616.91 (170.05–653.70)
ACT, unit	15.81 ± 4.59 (14.19–17.42)
AQLQ, unit	5.55 ± 1.23 (5.11–6.00)
mMRC, unit	1.39 ± 0.95 (1.05–1.72)
SNOT22, unit	31.10 ± 19.17 (24.24–37.96)
FVCpre, %pr	92.55 ± 23.87 (84.14–100.95)
FEV1pre, %pr	74.97 ± 23.43 (66.72–83.21)
FEV1/FVCpre, %	65.12 ± 12.51 (60.72–69.52)

BMI: Body Mass Index; Eos: eosinophils; IgE: immunoglobulin E; ACT: Asthma Control Test; AQLQ: Asthma Quality of Life Questionnaire; mMRC: Modified Medical Research Council Dyspnea Scale; SNOT22: Sino-Nasal Outcome Test 22; FVCpre: forced vital capacity, pre-bronchodilator; FEV1pre: forced expiratory volume in 1 s, pre-bronchodilator, %pr: percent of predicted value; CI: confident interval; SD: standard deviation; CI: confidence interval (95% lower–95% upper).

**Table 2:** Subjects’ pulmonary function tests.

Variable	Visit 1	N V1	Visit 2	N V2	Visit 3	N V3	*p* V1–V2	*p* V1–V3	*p* V2–V3
VCpre, L ± SD	2.99 ± 1.02	31	3.25 ± 0.97	25	3.59 ± 0.88	18	**0.014**	**0.018**	0.854
VCpre, %pr ± SD	93.06 ± 22.57	31	100.72 ± 22.46	25	104.61 ± 24.95	18	0.052	**0.013**	0.517
FVCpre, L ± SD	2.86 ± 0.93	31	3.08 ± 0.96	26	3.55 ± 0.81	18	**0.004**	**0.002**	0.071
FVCpre, %pr ± SD	92.55 ± 23.87	31	98.65 ± 22.51	26	106.78 ± 26.46	18	0.106	**0.001**	0.053
FVCpost, L ± SD	3.05 ± 1.05	31			3.69 ± 0.88	17		**0.007**	
FVCpost, %pr ± SD	97.90 ± 22.60	31			110.71 ± 24.53	17		**0.003**	
FEV1pre, L ± SD	1.84 ± 0.64	31	2.04 ± 0.73	26	2.37 ± 0.74	18	**0.003**	**0.002**	0.052
FEV1pre, %pr ± SD	74.97 ± 23.43	31	82.96 ± 26.57	26	88.89 ± 31.41	18	**0.041**	**0.003**	0.030
FEV1post, L ± SD	2.04 ± 0.75	31			2.59 ± 0.87	17		**0.005**	
FEV1post, %pr ± SD	82.58 ± 24.27	31			95.82 ± 31.89	17		**0.001**	
FEV1\FVCpre, % ± SD	65.12 ± 12.51	31	64.43 ± 19.03	26	67.10 ± 15.77	18	0.367	0.054	0.459
FEV1\FVCpost, % ± SD	67.63 ± 12.95	31			68.97 ± 16.78	16		**0.021**	

%pr: percent of the predicted value; pre: pre-bronchodilator; post: post-bronchodilator; VC: vital capacity; FVC: forced vital capacity; FEV1: forced expiratory volume in 1 s; L: liter; SD: standard deviation; N: number of subjects; *p*: *p* value.

**Table 3:** Subjects’ static lung volume and other indices of function tests.

Variable	Visit 1	N V1	Visit 2	N V2	Visit 3	N V3	*p* V1–V2	*p* V1–V3	*p* V2–V3
TLCpre, L ± SD	5.69 ± 1.44	30	5.80 ± 1.53	26	6.35 ± 1.18	18	0.220	0.963	0.284
TLCpre, %pr ± SD	105.37 ± 14.90	30	108.35 ± 19.05	26	111.94 ± 13.96	18	0.224	0.897	0.301
TLCpost, L ± SD	5.80 ± 1.36	30			6.27 ± 1.22	17		0.980	
TLCpost, %pr ± SD	108.10 ± 17.85	30			111.41 ± 13.46	17		0.940	
RVpre, L ± SD	2.77 ± 0.89	30	2.75 ± 1.00	26	2.84 ± 0.75	18	0.771	0.080	0.064
RVpre, %pr ± SD	136.33 ± 41.22	30	138.12 ± 48.23	26	143.06 ± 34.70	18	1.000	0.132	0.378
RVpost, L ± SD	2.73 ± 0.86	30			2.59 ± 0.73	17		0.130	
RVpost, %pr ± SD	135.60 ± 44.04	30			132.53 ± 33.87	17		0.159	
RV/TLC, % ± SD	48.75 ± 10.08	30	46.81 ± 10.61	26	44.63 ± 8.97	17	0.353	0.609	0.066
PEFpre, L/s ± SD	5.25 ± 1.90	31	5.66 ± 1.93	26	6.43 ± 1.99	18	**0.018**	0.067	0.109
PEFpre, %pr ± SD	76.13 ± 26.34	23	89.35 ± 23.06	20	96.65 ± 31.39	17	**0.017**	**0.039**	0.104
PEFpost, L/s ± SD	5.82 ± 2.17	30			6.94 ± 2.19	16		0.058	
PEFpost, %pr ± SD	83.91 ± 22.44	23			104.60 ± 31.00	15		**0.012**	
FEF25\75 pre, L/s ± SD	0.94 ± 0.62	31	1.25 ± 0.87	25	1.58 ± 1.03	17	0.066	**0.001**	0.130
FEF 25\75 pre, %pr ± SD	34.68 ± 22.56	31	45.32 ± 27.51	25	50.06 ± 29.14	17	0.106	**0.004**	0.231
FEF25\75 post, L/s ± SD	1.23 ± 0.81	30			2.03 ± 1.25	15		**0.015**	
FEF 25\75 post, %pr ± SD	43.37 ± 25.77	30			63.87 ± 33.67	15		**0.035**	
sRAW, cmH_2_O/s ± SD	5.73 ± 5.50	29	5.55 ± 4.49	25	4.60 ± 3.46	18	0.390	0.013	0.289
sRAW, %pr ± SD	131.14 ± 24.93	29	128.79 ± 28.85	25	126.88 ± 19.49	18	0.220	0.040	0.170
eNO, ppb ± SD	40 ± 48	26	20 ± 19	18	32 ± 47	14	0.069	0.217	0.432

TLC: total lung capacity; Rv: residual volume; PEF: peak expiratory flow; FEF25–75: forced expiratory flow at 25–75% of pulmonary volume; sRAW: specific airway resistance; eNO: exhaled nitric Oxide; pre: pre-bronchodilation; post: post-bronchodilation; %pr: percentage of predicted value; L: liters; L/s: liters per second; cmH_2_O/s: centimeters of water per second; SD: standard deviation; *p*: *p* value.

**Table 4:** Key variables in the forced oscillometry technique.

Variable	Visit 1	N V1	Visit 2	N V2	Visit 3	N V3	*p* V1–V2	*p* V1–V3	*p* V2–V3
Rrs5 tot, Zscore ± SD	1.11 ± 1.17	30	1.08 ± 1.62	26	0.80 ± 2.13	20	0.679	0.623	0.325
Rrs5 tot, %pr ± SD	144.31 ± 54.57	30	149.52 ± 76.73	26	149.81 ± 102.03	20	0.493	0.860	0.734
R5-19, cmH_2_O/(L/s) ± SD	0.48 ± 0.71	30	0.77 ± 0.73	26	0.65 ± 0.78	20	**0.009**	0.327	0.468
Xrs5 tot, cmH_2_O/(L/s) ± SD	−1.95 ± 1.97	30	−2.33 ± 2.11	26	−1.70 ± 1.74	20	0.819	0.738	0.580
Xrs5 tot, Zscore ± SD	−1.01 ± 2.93	30	−1.41 ± 3.56	26	−0.50 ± 3.36	20	0.954	0.768	0.671
Xrs5 tot, %pr ± SD	145.55 ± 118.73	30	165.49 ± 141.57	26	137.52 ± 145.42	20	0.909	0.738	0.609
AX, cmH_2_O/(L/s) ± SD	6.23 ± 5.03	30	7.10 ± 6.20	26	7.73 ± 11.61	20	0.954	0.651	0.495
AX, Zscore ± SD	−0.20 ± 2.48	30	0.11 ± 2.23	26	0.01 ± 2.27	20	0.954	0.623	0.442
AX, %pr ± SD	181.49 ± 176.71	30	221.21 ± 242.19	26	228.66 ± 272.83	20	0.932	0.515	0.523
Fres, cmH_2_O/(L/s) ± SD	14.16 ± 4.55	30	15.09 ± 6.15	26	14.68 ± 6.84	20	0.966	0.709	0.832
Fres, Zscore ± SD	0.24 ± 1.59	30	0.37 ± 1.80	26	0.41 ± 1.81	20	0.954	0.829	0.640
Fres, %pr ± SD	113.64 ± 38.64	30	120.26 ± 52.70	26	122.43 ± 61.02	20	0.797	0.738	0.799
EFL Xrs, cmH_2_O/(L/s) ± SD	1.26 ± 2.39	30	1.69 ± 2.66	26	0.87 ± 1.73	20	0.977	0.891	0.523

Rrs5: respiratory resistance at 5 Hz; Xrs5: total reactance at 5 Hz; AX: area under the reactance curve; Fres: resonant frequency; EFL Xrs: expiratory flow limitation; Zscore: Z-score; %pr: percentage of predicted value; cmH_2_O/(L/s): centimeters of water per liter per second; SD: standard deviation; *p*: *p* value.

**Table 5:** Prevalence of small airways disease assessed using various methods.

Parameter	V1 (%)	V2 (%)	V3 (%)	*p* Value
FVC, %pr < 80	41.94%	22.58%	22.58%	**0.0017**
SVC, %pr < 80	32.26%	19.35%	19.35%	**0.0274**
SVC–FVC, L < 0.100	51.61%	38.71%	38.71%	**0.0071**
FEF25/75, %pr < 80	93.55%	67.74%	67.74%	**<0.0001**
RV, %pr > 120	64.52%	51.61%	51.61%	0.0873
RV/TLC > 0.40	70.97%	67.74%	67.74%	0.5094
sRAW, %pr > 120	45.16%	35.48%	35.48%	0.2475
Rrs5 tot pre Z score > 1.645	22.58%	29.03%	29.03%	0.6142
Xrs5 tot pre Z score < −1.645	22.58%	29.03%	29.03%	0.6142
AX Z score < −1.645	16.13%	16.13%	16.13%	1.0000
Fres Z score < −1.645	6.45%	9.68%	9.68%	0.7706

FVC: forced vital capacity; FEV1: forced expiratory volume in 1 s, FEF25–75: forced expiratory flow at 25–75% of pulmonary volume; sRAW: specific airway resistance; Rrs5: respiratory resistance at 5 Hz; Xrs5: total reactance at 5 Hz; AX: area under the reactance curve; Fres: resonant frequency; EFL Xrs: expiratory flow limitation; Zscore: Z-score; %pr: percentage of predicted value; cmH_2_O/(L/s): centimeters of water per liter per second; SD: standard deviation.

## Data Availability

Data are available from the corresponding author.

## Supplementary Materials

The following supporting information can be downloaded at: https://www.mdpi.com/article/10.3390/jcm13195883/s1, Table S1: Demographic Characteristics and Comorbidities of the Study Population.

## Abbreviations

6MWT	Six-Minute Walk Test
ACT	Asthma Control Test
ANOVA	Analysis of variance
AQLQ	Asthma Quality of Life Questionnaire
ATS	American Thoracic Society
AX	Area of reactance
BD	Bronchodilation
COPD	Chronic obstructive pulmonary disease
ERS	European Respiratory Society
FeNO	Fraction of exhaled nitric oxide
FEF25–75%	Forced expiratory flow at 25–75%
FEV1	Forced expiratory volume in the first second
FOT	Forced oscillometry technique
Fres	Frequency of resonance
FVC	Forced vital capacity
GINA	Global Initiative for Asthma
HRCT	High-resolution computerized tomography
ICS	Inhaled corticosteroids
IgE	Immunoglobulin E
IL4α	Interleukin 4 receptor alpha
IL5	Interleukin 5
IL5Rα	Interleukin 5 receptor alpha
IOS	Impulse oscillometry technique
LABA	Long-acting beta ggonists
mMRC	Modified Medical Research Council Dyspnea Scale
MBNW	Multiple breath nitrogen washout
MRI	Magnetic resonance imaging
PET	Positron emission tomography
RV	Residual volume
SBNW	Single breath nitrogen washout
SAD	Small airway dysfunction
SF-12	12-Item Short Form Health Survey
SNOT22	Sino-Nasal Outcome Test-22
SPECT	Single-photon emission computed tomography
SVC	Slow vital capacity
TSLP	Thymic stromal lymphopoietin
TLC	Total lung capacity
X5	Reactance at 5 hertz
ΔX5in-esp	Difference in X5 between inspiration and expiration
R5–R19/20	Resistance at 5–19/20 hertz
