# Supplementary material for: Real-World Efficacy of Biological Therapies in Severe Asthma: A Focus on Small Airways
Source: J Clin Med. 2024 Oct 2;13(19):5883. doi: 10.3390/jcm13195883 (PMC11477072; doi:10.3390/jcm13195883)
Supplement: Supplementary file 1 [file jcm-13-05883-s001.zip › jcm-3193256-supplementary.pdf]

Supplementary Materials

This prospective observational study enrolled participants from July 2021 to March 2024, targeting patients with uncontrolled severe asthma managed at the Respiratory Disease Clinic, Tor Vergata University Hospital, Rome.

This was in independent no profit study. The study adhered to the Helsinki Declaration and received ethical approval on July 7, 2021, by the Independent Ethics Committee, STUDY PROTOCOL Severe Asthma MDB, CLINICAL TRIALS REGISTRY 132.21 P.U.OSS.

Inclusion and Exclusion Criteria

Inclusion criteria included patients over 18 years old diagnosed with severe asthma as per 2019 GINA guidelines (1), stable comorbidities not affecting asthma control, eligibility for biological therapy, and signed informed consent . Patients previously treated with biological agents were eligible. Exclusion criteria were under 18 years of age, active oncological diseases, psychiatric comorbidities, pregnancy, and inadequate availability for follow-up visits.

Definition of severe asthma

Severe asthma was defined according to GINA 2019 (1) as asthma that remains uncontrolled despite adherence to maximal optimized therapy and treatment of contributory factors, or that worsens when high-dose treatment is reduced. Uncontrolled asthma was defined by the presence of one or both of the following (1):

- Poor symptom control (e.g., frequent symptoms or reliever use, activity limitations due to asthma, or night-time awakenings caused by asthma)
- Frequent exacerbations (≥2/year) requiring oral corticosteroids (OCS), or serious exacerbations (≥1/year) requiring hospitalization

Maximal therapy for asthma was defined as the highest level of pharmacological treatment prior to the introduction of biological agents. This included high-dose inhaled corticosteroids (ICS), administered at the maximum recommended dose to control airway inflammation, and long-acting beta-agonists (LABA), combined with ICS to provide sustained bronchodilation. Additional therapies were included as required, such as long-acting muscarinic antagonists (LAMA) to enhance bronchodilation in patients with persistent symptoms, leukotriene receptor antagonists (LTRA) prescribed to manage allergic components of asthma when appropriate.

Maximal therapy also involved the identification and treatment of contributing factors such as allergies, obesity, gastroesophageal reflux disease (GERD), and other comorbidities. Patients were required to adhere to this regimen before considering the use of biological therapies.

The biological agents were prescribed based on clinical decisions and in accordance with AIFA recommendations. Specifically Omalizumab was prescribed in patients with severe persistent allergic asthma who have a documented allergic asthma with positive skin test or in vitro reactivity to perennial allergens, a history of frequent exacerbations despite high-dose inhaled corticosteroids (ICS) and long-acting beta-agonists (LABA) treatment, and total serum IgE levels between 30 and 1500 IU/mL and FEV1 lower than 80%. Dupilumab was prescribed for patients with severe asthma with type 2 inflammation, defined by evidence of elevated eosinophils (≥150 cells/μL) or FeNO (≥25 ppb). Mepolizumab was prescribed for patients with severe eosinophilic asthma who have an elevated blood eosinophil count, typically ≥150 cells/μL at screening or ≥300 cells/μL in the past 12 months. Benralizumab was prescribed for patients with severe eosinophilic asthma who have high eosinophil levels, typically ≥300 cells/μL.
The protocol did not influence the choice of biological agents. If a patient was eligible for more than one drug, the decision was made at the discretion of the clinician.

Protocol

Evaluations were conducted at baseline (prior to initial drug administration) and at subsequent intervals of 1, 3, months post-therapy initiation, covering three assessment points (T0-T1-T3).
At Visit 1-T0, consent was obtained and parameters such as comorbidities, exacerbations in the prior year, blood eosinophils, total IgE levels, and a series of pulmonary function tests (including pre- and post-bronchodilation (BD) spirometry, FOT, exhaled nitric oxide fraction (FeNO), and a six-minute walk test (6MWT)) were assessed. Questionnaires (Modified Medical Research Council Dyspnea Scale – mMRC; Asthma Control Test – ACT; Sino-Nasal Outcome Test-22 - SNOT22; 12-Item Short Form Health Survey - SF-12) were also administered. These variables were reviewed at each follow-up visit.

Study Timelines and Examinations

Visit 1 (T0 – Study Entry)

At the time of study entry, consent was obtained, and parameters such as comorbidities, exacerbations in the prior year, blood eosinophils, and total IgE levels were recorded.
Patients then completed the following questionnaires: the modified Medical Research Council (mMRC) scale, the Asthma Control Test (ACT), the Asthma Quality of Life Questionnaire (AQLQ), and the Sino-Nasal Outcome Test (SNOT-22).
Pulmonary function tests were performed, including the slow maneuver, the forced spirometry pre- and post-bronchodilation, body plethysmography, and diffusing capacity for carbon monoxide

(DLCO). The Forced Oscillometry Technique (FOT) was performed before and after bronchodilation, as the first test in the series. The exhaled fraction of bronchial nitric oxide (FeNO) and the 6-minute walk test (6MWT) were also conducted before bronchodilation.

Visit 2 (T1 – After 1 Month)

Visit 2, conducted one month after the start of therapy, was similar to Visit 1, but spirometry and FOT were performed without bronchodilation.

Visit 3 (T3 – After 3 Months)

Visit 3, conducted three months after therapy initiation, followed the same procedures as Visit 1, with all tests performed, including pre- and post-bronchodilation spirometry.

Questionnaires

Asthma Control Test (ACT): The ACT is a simple, five-question tool used to assess asthma control over the past four weeks. It evaluates symptoms, use of rescue medication, and the impact of asthma on daily activities. Scores range from 5 to 25, with higher scores indicating better asthma control. A score of 19 or below indicates poorly controlled asthma (2,3). The Minimal Clinical Importance Difference (MCID) for the ACT is generally considered to be 3 points (4).

Sino-Nasal Outcome Test (SNOT-22): This is a 22-item questionnaire used to assess the impact of chronic rhinosinusitis on a patient's quality of life, including physical, functional, and emotional consequences. Each item is scored from 0 (no problem) to 5 (worst imaginable problem), with a total score range of 0 to 110 (5). Validated in Italian language (6). The MCID for SNOT-22 is 8.9 points(5).

Modified Medical Research Council (mMRC) Dyspnea Scale: This tool measures the severity of breathlessness in patients with respiratory diseases such as COPD and asthma. It ranges from 0 (no dyspnea except with strenuous exercise) to 4 (too breathless to leave the house or when dressing)(7). For the mMRC, an improvement of 1 point is considered to represent a clinically significant change(8,9).

Asthma Quality of Life Questionnaire (AQLQ): The AQLQ assesses the impact of asthma on a patient's quality of life across four domains: symptoms, activity limitation, emotional function, and environmental stimuli. It includes 32 items, and higher scores reflect worse quality of life. This tool is widely used in both clinical trials and clinical practice (10,11). Translated in several languages (12). All the questionnaires were administered via interview. The questionnaires are available in the corresponding references. The MCID for the AQLQ is 0.5 points on a 7-point scale(10).

Pulmonary Function Tests

Pulmonary function testing was performed using either the Carefusion MasterScreen PFT (Pulmonary Function Testing) System or the Cosmed Q-Box Body Plethysmography. Each patient consistently used the same apparatus for all pulmonary function tests throughout the study.
Spirometry and plethysmography were conducted in accordance with internal guidelines (13,14).
Slow and forced manoeuvres were conducted with the patient seated in an upright position, wearing a nose clip to prevent air leakage through the nose. A mouthpiece connected to the spirometer was used, and the patient was instructed to maintain a tight seal around it. For the slow manoeuvre, the patient performed a slow vital capacity (SVC) manoeuvre by inhaling fully and exhaling slowly and steadily to measure lung volumes. For the forced manoeuvre, the patient inhaled deeply and exhaled forcefully and as quickly as possible to measure forced expiratory volume in one second (FEV1) and forced vital capacity (FVC).
Body plethysmography was performed with the patient seated inside an airtight chamber, again wearing a nose clip. The patient breathed through a mouthpiece, and changes in pressure inside the chamber were used to measure static lung volumes, such as total lung capacity (TLC) and residual volume (RV).
At least three acceptable manoeuvres were performed for both spirometry and plethysmography, according to standard criteria. For acceptability, each manoeuvre had to show no hesitation or early termination, with a satisfactory start and smooth exhalation. For reproducibility, the difference between the two largest values for FVC and FEV1 had to be within 150 mL. Similarly, lung volume measurements for plethysmography needed to meet reproducibility standards (13,14).
The Forced Oscillometry Technique was performed using the ResTech Resmon Pro Full (Version 3), a non-invasive device that applies small pressure oscillations during spontaneous breathing to evaluate respiratory resistance (R) and reactance (X). Initially, version 2 was used, which was later converted to version 3. The patient was seated in an upright position, breathing through a mouthpiece with a nose clip applied, and hands placed on the cheeks to prevent movement that could interfere with measurements. At least, ten breaths were recorded in three different sessions to ensure consistency and accuracy of the results.
Exhaled nitric oxide levels were assessed using the Bosch Healthcare Vivatmo Pro device. This device measures the fraction of exhaled nitric oxide (FeNO). Patients were seated upright and instructed to exhale slowly and steadily through a mouthpiece after taking a deep breath, following the device's prompts to ensure consistent flow and volume. FeNO levels were recorded in parts per billion (ppb). At least two measurements were taken for each patient to ensure reliability(15,16).

Statistical Analysis

Data were collected in a de-identified database, and the following steps were carried out for statistical analysis:

Normality of Continuous Variables: Continuous variables were tested for normality using the Shapiro-Wilk test (or another test used), ensuring that appropriate statistical methods were applied based on the distribution of the data. In cases where normality was not assumed, non-parametric tests, such as the Wilcoxon signed-rank test, were used for paired comparisons.

Sample Size Calculation: A formal sample size calculation was not conducted prior to the study. This decision was based on the limited availability of studies in the literature for reference, as well as the exploratory nature of the analysis. However, the sample size was determined based on the available data, taking into consideration the constraints of the study population. We acknowledge that this might impact the statistical power, and this limitation is discussed in the study's limitations section.

Handling Missing Data: Prior to any statistical tests, missing data were addressed. Incomplete observations were excluded from the regression analysis. Additionally, multiple imputation was applied in cases where it was assumed that the missing data depended on other observed variables (Missing At Random, MAR). Sensitivity analyses were also conducted to assess how the results might change under different assumptions about the missing data.

Descriptive Analysis: Descriptive statistics were performed to provide an initial overview of the data, calculating the mean, median, standard deviation, and interquartile range for the parameters of interest, categorized by treatment group and visit. Frequency tables and graphical representations, such as bar plots and box plots, were used to visualize the distribution of categorical data.

Inferential Analysis: To evaluate treatment response, Wilcoxon signed-rank tests were performed for paired comparisons when analyzing non-parametric, repeated data. Multiple regression analyses were conducted to determine predictors of treatment response based on baseline variables, while accounting for covariates through an analysis of covariance (ANCOVA) when necessary.

Evaluation of Treatment Effects: Repeated measures ANOVA was used to compare the means of multiple groups over time. For time-to-event outcomes, survival analysis techniques were applied, especially in cases with censored data. Univariate correlations were examined using Pearson’s product moment-correlation to explore associations between variables.

Tools and Software: All statistical analyses were conducted using Python, with libraries such as Pandas and SciPy, and GraphPad Prism 9. Data are presented as mean ± standard deviation (SD) unless otherwise specified. A p-value of <0.05 was considered statistically significant for all analyses.

Table S1. Demographic Characteristics and Comorbidities of the Study Population.

Parameter	*Frequency (Percentage)*
Female, n (%)	21 (67.74)
Male, n (%)	10 (32.26)
Current Smoking , n (%)	1 (3.23)
Smoking Exposure, n (%)	10 (32.26)
Allergies, n (%)	22 (70.97)
Gastroesophageal Reflux Disease (GERD), n (%)	17 (54.84)
Polyposis, n (%)	15 (48.39)
Dermatitis, n (%)	4 (12.90)
Orticaria, n (%)	4 (12.90)
Bronchiectasie, n (%)	10 (32.26)
Rhinitis/Sinusitis, n (%)	8 (25.81)
Rheumatological diseases, n (%)	8 (25.81)
Other diseases, n (%)	24 (77.42)

References

1. GINA Report, Global Strategy for Asthma Management and Prevention. 2019; Available from: https://ginasthma.org/wp-content/uploads/2019/06/GINA-2019-main-report-June-2019-wms.pdf
2. Nathan RA, Sorkness CA, Kosinski M, Schatz M, Li JT, Marcus P, et al. Development of the asthma control test: a survey for assessing asthma control. J Allergy Clin Immunol. 2004 Jan;113(1):59–65.
3. Schatz M, Kosinski M, Yarlas AS, Hanlon J, Watson ME, Jhingran P. The minimally important difference of the Asthma Control Test. Journal of Allergy and Clinical Immunology [Internet]. 2009 Oct [cited 2024 Sep 21];124(4):719-723.e1. Available from: https://linkinghub.elsevier.com/retrieve/pii/S0091674909012457
4. Bonini M, Di Paolo M, Bagnasco D, Baiardini I, Braido F, Caminati M, et al. Minimal clinically important difference for asthma endpoints: an expert consensus report. Eur Respir Rev [Internet]. 2020 Jun 30 [cited 2024 Jan 3];29(156):190137. Available from: http://err.ersjournals.com/lookup/doi/10.1183/16000617.0137-2019
5. Hopkins C, Gillett S, Slack R, Lund VJ, Browne JP. Psychometric validity of the 22-item Sinonasal Outcome Test. Clin Otolaryngol. 2009 Oct;34(5):447–54.
6. Mozzanica F, Preti A, Gera R, Gallo S, Bulgheroni C, Bandi F, et al. Cross-cultural adaptation and validation of the SNOT-22 into Italian. Eur Arch Otorhinolaryngol. 2017 Feb;274(2):887–95.
7. Mahler DA, Rosiello RA, Harver A, Lentine T, McGovern JF, Daubenspeck JA. Comparison of Clinical Dyspnea Ratings and Psychophysical Measurements of Respiratory Sensation in Obstructive Airway Disease [1–4]. Am Rev Respir Dis [Internet]. 1987 Jun [cited 2024 Sep 21];135(6):1229–33. Available from: http://www.atsjournals.org/doi/10.1164/arrd.1987.135.6.1229
8. Cazzola M, MacNee W, Martinez FJ, Rabe KF, Franciosi LG, Barnes PJ, et al. Outcomes for COPD pharmacological trials: from lung function to biomarkers. Eur Respir J. 2008 Feb;31(2):416–69.
9. Araújo Oliveira AL, Andrade L, Marques A. Minimal clinically important difference and predictive validity of the mMRC and mBorg in acute exacerbations of COPD. In: Physiotherapists [Internet]. European Respiratory Society; 2017 [cited 2024 Sep 22]. p. PA4705. Available from: http://erj.ersjournals.com/lookup/doi/10.1183/1393003.congress-2017.PA4705
10. Juniper EF, Guyatt GH, Willan A, Griffith LE. Determining a minimal important change in a disease-specific Quality of Life Questionnaire. J Clin Epidemiol. 1994 Jan;47(1):81–7.
11. Juniper EF, Guyatt GH, Epstein RS, Ferrie PJ, Jaeschke R, Hiller TK. Evaluation of impairment of health related quality of life in asthma: development of a questionnaire for use in clinical trials. Thorax. 1992 Feb;47(2):76–83.
12. AQLQ https://www.thoracic.org/members/assemblies/assemblies/srn/questionaires/aqlq.php.
13. Miller MR, Hankinson J, Brusasco V, Burgos F, Casaburi R, Coates A, et al. Standardisation of spirometry. Eur Respir J. 2005 Aug;26(2):319–38.
14. Wanger J, Clausen JL, Coates A, Pedersen OF, Brusasco V, Burgos F, et al. Standardisation of the measurement of lung volumes. Eur Respir J [Internet]. 2005 Sep [cited 2022 Feb 20];26(3):511–22. Available from: http://erj.ersjournals.com/lookup/doi/10.1183/09031936.05.00035005
15. Dweik RA, Boggs PB, Erzurum SC, Irvin CG, Leigh MW, Lundberg JO, et al. An Official ATS Clinical Practice Guideline: Interpretation of Exhaled Nitric Oxide Levels (F E NO) for Clinical Applications. Am J Respir Crit Care Med [Internet]. 2011 Sep 1 [cited 2024 Apr 9];184(5):602–15. Available from: https://www.atsjournals.org/doi/10.1164/rccm.9120-11ST

16. Korn S, Wilk M, Voigt S, Weber S, Keller T, Buhl R. Measurement of Fractional Exhaled Nitric Oxide: Comparison of Three Different Analysers. Respiration [Internet]. 2020 [cited 2024 Sep 21];99(1):1–8. Available from: https://karger.com/RES/article/doi/10.1159/000500727
